# The Impact of Emergency Interventions and Patient Characteristics on the Risk of Heart Failure in Patients with Nontraumatic OHCA

**DOI:** 10.1155/2019/6218389

**Published:** 2019-12-16

**Authors:** Cheng Hsu Chen, Chih-Yu Chang, Mei-Chueh Yang, Jr-Hau Wu, Ching-Hui Liao, Chih-Pei Su, Yu-Chih Chen, Shinn-Ying Ho, Cheng-Chieh Huang, Tsung-Han Lee, Wen-Liang Chen, Chu-Chung Chou, Yan-Ren Lin

**Affiliations:** ^1^Department of Emergency Medicine, Changhua Christian Hospital, Changhua, Taiwan; ^2^Department of Biological Science and Technology, National Chiao Tung University, Hsinchu, Taiwan; ^3^Department of Nursing, Changhua Christian Hospital, Changhua, Taiwan; ^4^School of Medicine, Kaohsiung Medical University, Kaohsiung, Taiwan; ^5^School of Medicine, Chung Shan Medical University, Taichung, Taiwan

## Abstract

**Background:**

Since out-of-hospital cardiac arrest- (OHCA-) related dysfunction (ischemic/reperfusion injury and inflammatory response) might result in long-term impairment, we suspect that new-onset heart failure might be common in long-term survivors. However, these relationships had not been well addressed, and we aimed to analyze the impact of emergency interventions and patient characteristics on the risk of new-onset heart failure in patients with nontraumatic OHCA.

**Methods:**

The Taiwanese government healthcare database contains data for 49,101 nontraumatic OHCA adult patients from 2011-2012, which were analyzed in this study. Nontraumatic OHCA patients who survived to the intensive care unit (ICU) were included as the study group (*n* = 7,321). Matched patients (*n* = 21,963) were recruited as a comparison group. Patients with any history of heart failure or cardiac arrest were not included in either group. All patients were followed-up for 6 months for the identification of new-onset heart failure. Adjustments were made for demographics, age, emergency interventions, and comorbidities as potential risk factors.

**Results:**

In all, 3.84% (*n* = 281) of OHCA patients suffered new-onset heart failure, while only 1.24% (*n* = 272) of matched patients in the comparison group suffered new-onset heart failure. Strong risk factors for heart failure were age (60–75 years, HR: 11.4; 95% CI: 9–14.4), medical history (myocardial infarction, HR: 2.47; 95% CI: 2.05–2.98 and cardiomyopathy, HR: 2.94; 95% CI: 1.45–5.94), and comorbidities during hospitalization (ischemic heart disease, HR: 4.5; 95% CI: 3.46–5.86). Only extracorporeal membrane oxygenation (ECMO) decreased the risk of heart failure. Most (53.6%) heart failure events occurred within 60 days after OHCA.

**Conclusion:**

An age from 61 to 75 years, a history of myocardial infarction or cardiomyopathy, and ischemic heart disease or infection as comorbidities occurring during hospitalization were strong risk factors for new-onset heart failure in OHCA patients. However, ECMO could decrease this risk. More importantly, most heart failure events occurred within 60 days after OHCA.

## 1. Introduction

Although some nontraumatic out-of-hospital cardiac arrest (OHCA) patients can initially achieve restoration of spontaneous circulation (ROSC) after resuscitation, the overall survival rate of OHCA ranges only from 5.7% to 8.3% [1–4]. Even among OHCA patients discharged from the hospital, the one-year survival rate is only from 7.7% to 11.5% [1–3,5]. Some major risk factors, including unhealed underlying diseases, arrest-related hypoxia injury, and new-onset postresuscitation comorbidities, are considered to be responsible for this poor outcome [6–8]. Furthermore, new-onset postresuscitation comorbidities, which might interact with essential diseases or cause sickness independently, can be life-threatening and difficult to prevent [9–14].

Among such postresuscitation comorbidities, new-onset heart failure might be the most important factor associated with the cardiopulmonary function and hemodynamics of OHCA patients [8]. Diseases of cardiac origin (i.e., acute coronary syndrome and cardiac arrhythmia) are some of the most common etiologies of OHCA (accounting for 60% to 70%); myocardial dysfunction is also common (up to 66%) during the early postresuscitation period [15, 16]. Therefore, we suspect that new-onset heart failure might be very predominant among OHCA survivors. Although a few animal studies have tried to identify the risk factors (including ischemia/reperfusion injury, systemic inflammatory response, and catecholamine surge) of postresuscitation heart failure in humans, this information has not been well addressed, and there is a lack of relevant long-term follow-up investigations [12, 17, 18]. Since some causes of this dysfunction (I/R injury, inflammatory response) might result in long-term impairment, we suspect that new-onset heart failure might be common in long-term survivors. More importantly, cardiac arrest-related complications and emergency interventions might also influence the occurrence of heart failure. However, these relationships had not been well addressed before this large population study. In this study, we aimed to analyze the impact of emergency interventions and patient characteristics on the risk of new-onset heart failure in patients with nontraumatic OHCA.

## 2. Methods and Materials

### 2.1. Data Source

Taiwan's Ministry of Health and Welfare (MOHW) established a Health and Welfare Data Center (HWDC), a data repository site that centralizes the NHIRD and about 70 other health-related databases for data management and analyses. The data that we used were obtained from the Taiwan's National Health Insurance Research Database (NHIRD). This program is centralized by the Health and Welfare Data Center (HWDC) and supported by the Taiwan's Ministry of Health and Welfare (MOHW). The NHIRD covers over 99% of Taiwan's population (over 22 million people). With the MOHW's permission, data from the NHIRD could be extracted and analyzed for the purpose of scientific research.

### 2.2. Ethical Approval

#### 2.2.1. Institutional Review Board (IRB) Permission

The protocol of this study was reviewed and approved by the IRB of Changhua Christian Hospital (permission code: 150117). To ensure that the data extracted from the NHIRD were used only for scientific research, our protocol, IRB permission, and output data were all supervised by the Taiwanese government.

#### 2.2.2. Patient Privacy Policy

To protect personal privacy, all patients' medical records were secondary data (deidentified). Moreover, the data were not allowed to be displayed if the specific search parameters yielded fewer than 3 patients (identification possible).

### 2.3. Study Setting and Population

**Figure 1:**
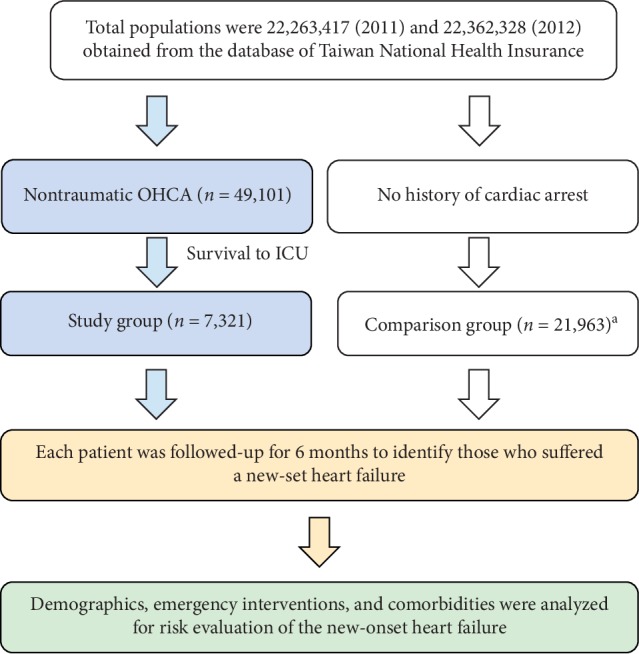
Selection flowchart of this study.

This was a retrospective cohort study. The study and comparison groups comprised patients with database records during the study period (January 2011 to December 2012). The selection flowchart is shown in Figure 1. All patients were followed-up for 6 months for the identification of those who suffered new-onset heart failure. In addition, the demographics, medical histories, emergency interventions, and comorbidities were analyzed according to three age groups (<60 years, 60–75 years, and >75 years) for the risk evaluation.

#### 2.3.1. Inclusion Criteria

*(1) Definition of Study Group*. Patients who met all of the following characteristics during the study period were included in the study group:

1. Patients had experienced their first nontraumatic OHCA and were diagnosed by emergency department (ED) physicians, with OHCA defined using the International Classification of Diseases, 9th revision, clinical modification (ICD-9-CM) codes 798–799 and 427.5
2. Patients had ever received resuscitation in the ED and survived to intensive care unit (ICU) admission

*(2) Definition of Comparison Group*. Patients in this group were randomly selected from the remaining patients in the NHIRD. They were matched to patients in the study group in terms of age, sex, and follow-up period. Three times as many patients were included in this group than in the study group.

*(3) Definition of Primary Outcome (New-Onset Heart Failure)*. New-onset heart failure was diagnosed according to the ICD-9-CM code 428.X.

#### 2.3.2. Exclusion Criteria

Patients with the following conditions were excluded from both groups:

1. Patients with any history of heart failure or cardiac arrest (traumatic/nontraumatic) before the study began
2. OHCA patients who did not receive postresuscitation care or evaluations
3. Pediatric patients (age <18 years)
4. Patients with OHCA related to trauma (major diagnosis including the following ICD-9-CM codes: 800–809, 810–819, 820–829, 830–839, 850–854, 860–862, 863–869, 900–904, 925–929, 940–949, and 950–957)
5. Patients with incomplete medical records
6. Patients who terminated their insurance during the study period

### 2.4. Study Protocol

In all, 7,321 OHCA patients (surviving to ICU admission) were included in the study group, and 21,963 patients were included in the comparison group. Among the study group, cardiac and noncardiac causing OHCA were identified. We followed-up all patients for 6 months to identify those who suffered new-onset heart failure. Moreover, this study considered and adjusted the hazard ratio (HR) depending on different variables between the two groups.

### 2.5. Data Analysis

Descriptive statistics, the Chi-squared test, the crude HR with Cox proportional hazards models, the log-rank test, and the Kaplan–Meier method were applied in this study. We used SAS (SAS Institute, Inc., Cary, NC, USA) to select patients. The SAS programming language and the results of the analysis were routinely checked by governmental database supervisors to ensure that all information was deidentified.

Descriptive statistics for independent variables were first determined. Demographics, including sex, age, economic level, geographic region and degree of residence urbanization, and medical history, including myocardial infarction, hypertension, diabetes mellitus, cardiomyopathy (congestive, constrictive, familial, idiopathic, infiltrative, obstructive, and nonspecific), chronic obstructive pulmonary disease (COPD) and asthma, chronic kidney disease (CKD), systemic lupus erythematosus (SLE), liver cirrhosis, atrial flutter/atrial fibrillation, sicca syndrome, and rheumatic arthritis, are reported as the number, percentage, or mean ± standard deviation (SD). Detailed information regarding how the economic levels, geographic regions, and degrees of urbanization were classified has been described in previous database studies [19, 20]. In addition, information on emergency interventions (for treating OHCA), including defibrillation, PCI, ECMO, and therapeutic hypothermia, are reported. New-onset comorbidities during hospitalization were mainly categorized as infection (i.e., respiratory tract infection, urinary tract infection, and septicemia), ischemic heart disease (i.e., acute coronary syndrome), and nonischemic heart disease (i.e., valvular heart disease, arrhythmia, cardiomyopathy, and myocarditis).

The Chi-squared test was used to determine differences in the patient characteristics and demographics between the study and comparison groups, as well as differences in the clinical features of patients with new-onset heart failure and those without heart failure. To analyze the association between heart failure and patient age, all patients were classified into three age groups (<60, 60–75, and >65 years). For these three age groups, crude HRs were calculated (for heart failure) using Cox proportional hazards models. Furthermore, to further evaluate the other potential impact factors, the HR was analyzed after adjusting demographics (model 1), medical history (model 2), emergency interventions (model 3), new-onset comorbidities during hospitalization (model 4), and all variables (model 5). The sensitivity analysis was also performed for patients admitted to ICU and those not admitted to ICU (for all OHCA patients and cardiac-caused OHCA patients). All the factors were considered in this analysis, including demographics, emergency interventions, and medical histories (atrial flutter and atrial fibrillation, hypertension, and diabetes mellitus).

Time-related factors associated with the occurrence of new-onset heart failure were also calculated. Heart failure-free survival curves were estimated using the Kaplan–Meier method and log-rank test. Among the patients with heart failure, the time from OHCA to the onset of heart failure was recorded and further divided into three periods (<60, 60–120, and 120–180 days). A *p* value <0.05 was considered statistically significant.

## 3. Results

### 3.1. Characteristics and Demographics of OHCA Patients

During the study period, 49,101 nontraumatic OHCA adult patients survived to ICU admission, 10,185 (20.7%) survived to ICU admission, and 3,767 (7.8%) survived to discharge. The final number of study patients (chosen according to the inclusion and exclusion criteria) was 7,321. The characteristics and medical history of patients in the study (*n* = 7,321) and comparison (*n* = 21,963) groups are presented in Table 1. Compared with patients in the comparison group, those in the study group had a lower economic level. Moreover, the incidence of myocardial infarction, hypertension, diabetes, cardiomyopathy, COPD and asthma, CKD, and liver cirrhosis was significantly higher among OHCA patients (all *p* < 0.001). In addition, 17.1% (*n* = 1255) of OHCA patients had received defibrillation, and 7.3% (*n* = 532) had received catheterization. Only 1.8% and 3.7% of OHCA patients received ECMO and hypothermia therapy, respectively. Nonischemic heart disease and infection were the two most common new-onset comorbidities during hospitalization. The cumulative incidence of heart failure is 3.84%.

### 3.2. Clinical Features of Patients Who Suffered New-Onset Heart Failure

Among the 7,321 OHCA patients, 363 patients (4.9%) suffered new-onset heart failure during the follow-up period. Their clinical features compared with those of the patients without heart failure are shown in Table 2. Patients older than 75 years accounted for the majority of patients with heart failure. Myocardial infarction, hypertension, and cardiomyopathy were significantly more common in heart failure patients (all *p* < 0.05). Additionally, significantly more patients with and without heart failure received defibrillation and PCI in the ED (both *p* < 0.05).

### 3.3. HRs for New-Onset Heart Failure in Different Age Groups

The covariate-adjusted HRs for new-onset heart failure were significantly higher in OHCA patients than in comparison patients (Table 3). Moreover, we found that a variety of HRs differed among the age groups. OHCA patients who are 60–75 years old had a higher covariate-adjusted HR (HR: 11.4, 95% CI: 9.0–14.4) than those who are <60 years old (HR: 5.6, 95% CI: 4.3–7.3) and >75 years old (HR: 10.7, 95% CI: 8.5–13.4). In addition, the risk of heart failure was much higher in cardiac-caused OHCA groups (*n* = 3,040, 41.5%). Among them, the crude HRs were significantly higher than comparison patients (Supplementary [Supplementary-material supplementary-material-1]).

### 3.4. Variables Influencing the Risk of New-Onset Heart Failure

The adjustments for likely influencing factors are presented in Table 4. The risks of heart failure were more predominant in the sensitivity analysis conducted on emergency interventions and medical histories (hypertension, atrial flutter/atrial fibrillation, and diabetes mellitus) (data not shown).

#### 3.4.1. Medical History

For all OHCA patients, myocardial infarction, hypertension, cardiomyopathy, and flutter/atrial fibrillation were strongly associated with the occurrence of heart failure. Myocardial infarction and cardiomyopathy had a significant impact in all age groups. Hypertension increased the risk more in those aged >75 years. Moreover, cardiomyopathy was the most powerful risk factor in those aged <60 years (HR: 4.62, 95% CI: 1.89–11.29).

#### 3.4.2. Emergency Interventions

In all age groups, patients who had ever required emergency PCI or defibrillation were at a higher risk of heart failure (model 3). While ECMO decreased the risk of heart failure (HR: 0.23, 95% CI: 0.07–0.73), therapeutic hypothermia did not.

#### 3.4.3. New-Onset Comorbidities during Hospitalization

New-onset ischemic heart disease occurring during hospitalization was the comorbidity with the most influence on the occurrence of heart failure (model 4). Furthermore, we found that ischemic heart disease strongly increased the risk of heart failure in patients aged <60 years (HR: 6.72, 95% CI: 4.34–10.42).

### 3.5. Heart Failure-Free Survival

**Figure 2:**
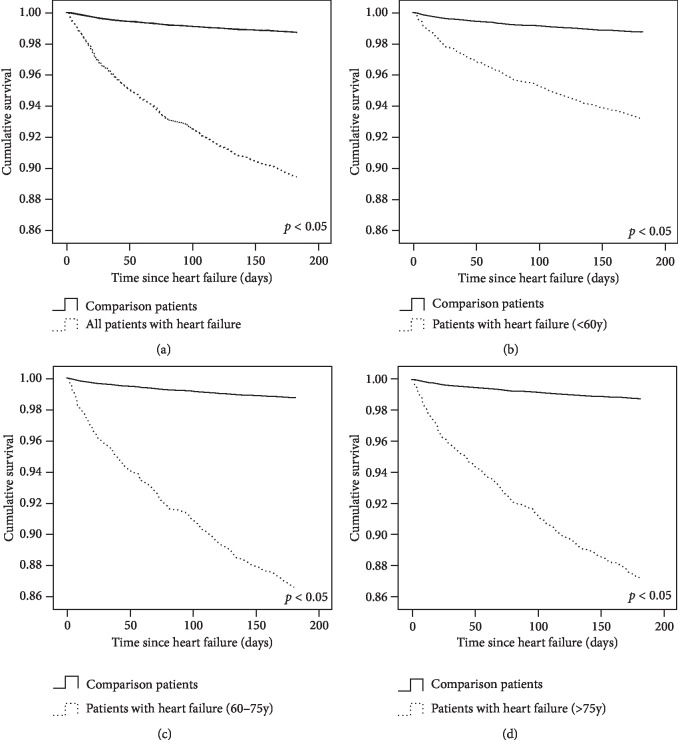
The heart failure-free survival curves of study and comparison patients during the follow-up period. The incidence of heart failure-free survival was significantly lower in OHCA patients than comparison patients (all age groups, *p* < 0.05). Heart failure occurred more quickly in patients aged 61–75 years (Figure 2(c)) than in those in the other age groups (Figures 2(a), 2(b), and 2(d)).

The heart failure-free survival curves of the study and comparison patients during the follow-up period are shown in Figure 2. The incidence of heart failure-free survival was significantly lower in OHCA patients than in comparison patients (all age groups, *p* < 0.05). Heart failure occurred more quickly in patients aged 61–75 years (Figure 2(c)) than in those in the other age groups (Figures 2(a), 2(b), and 2(d)).

### 3.6. Time between OHCA and Heart Failure Onset

**Figure 3:**
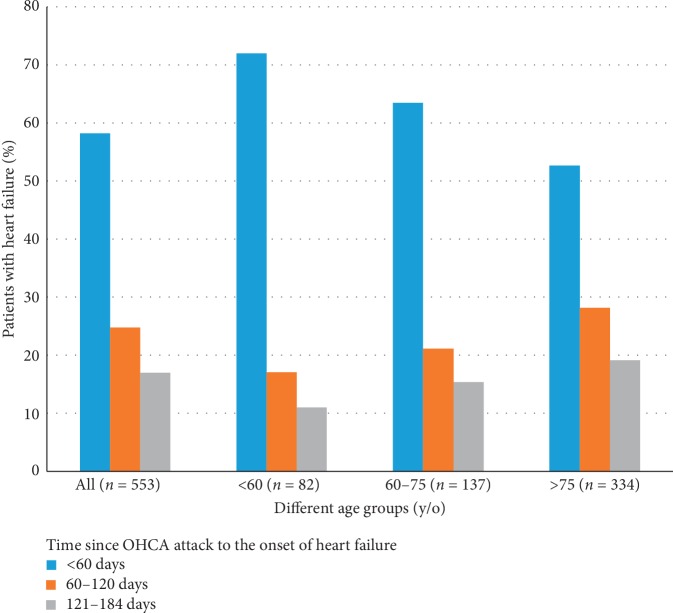
The time between OHCA and new-onset heart failure.

The time between OHCA and new-onset heart failure is shown in Figure 3. For all age groups, most (58.2%) heart failure events occurred within 60 days after OHCA, especially in patients younger than 60 years (<60 years: 72%, 60–75 years: 63.5%, and >70 years: 52.7%). In addition, a delayed onset (121–180 days) of heart failure was more common in those aged >75 years (19.2%) than in those aged <60 years (11%) and 61–75 years (15.3%).

## 4. Discussion

Myocardial dysfunction has been reported as a common comorbidity during the early postresuscitation period, occurring in up to 66% of ROSC patients [10]. This presentation would peak between 2 and 5 hours after CPR [6–9,11]. Myocardial dysfunction is usually induced by ischemia/reperfusion (I/R) injury, systemic inflammatory responses, and catecholamine surges [9]. Damage to the myocardium can cause low cardiac output, unstable hemodynamics, and even neurological impairment [9]. Despite this condition being critical, some studies have reported that it is a short-term comorbidity and could be reversible within 72 hours [9–11].

We found that the risk of new-onset heart failure was higher in long-term OHCA survivors than in comparison patients. The first key risk factor was age. Some previous studies have reported that the risk of heart failure increased in proportion with patient age [21, 22]. However, in this study, compared with the younger and older patients, patients aged 60 to 75 years showed the highest risk. One reason might be that cardiovascular disease (e.g., coronary artery disease) was more common in this age group [23–25]. However, heart failure normally increased with age, and our findings also need to consider potential survival bias.

The second key risk factor for new-onset heart failure was a history of myocardial infarction, hypertension, or cardiomyopathy. Our analysis showed that a history of diabetes mellitus, COPD/asthma, and CKD did not significantly increase the risk. Myocardial infarction and cardiomyopathy were the most powerful risk factors, increasing the risk in all age groups. We suspect that myocardial damage would be worse in OHCA patients with a history of myocardial infarction or a certain extent of myocardial stunning, which might result in poorer coronary perfusion during resuscitation or the postresuscitation period. This condition might further cause irreversible myocardial dysfunction and long-term heart failure [6, 7]. A history of cardiomyopathy was a risk factor only in patients aged less than 60 years. The major reason for this finding is that young patients with earlier gene presentation (e.g., sarcomere protein mutations) would have more severe mitral valve regurgitation and pulmonary artery wedge pressure [26–29]. We suspect that pulmonary hypertension and left ventricle outlet obstruction could be more predominant after resuscitation and thus increase the risk of heart failure. In addition, the heart failure itself includes several types of cardiomyopathies. However, we suspect that cardiomyopathy would be a strong risk factor and even be a confounding factor. Therefore, we did not exclude those patients and try to put them in our regression models.

The occurrence of new-onset comorbidities during hospitalization was the third key risk factor for heart failure; ischemic heart disease occurring during hospitalization significantly increased this risk. As the cardiac output, rhythms, vascular resistance, and contraction ability are unstable during the postresuscitation period, [6–8,10] new-onset diseases affecting the heart could independently increase the cardiovascular load and result in irreversible heart failure. Therefore, aggressive heart disease prophylaxis and treatment during the early postresuscitation period should be emphasized. In addition, in our adjusted models, infection increased the risk of new-onset heart failure in the long-term follow-up period. Some previous studies have also demonstrated that severe infection could increase OHCA-related mortality and impair hemodynamics during ICU admission [9, 30].

The final key factors were emergency interventions, and we found that emergency defibrillation and PCI both increased the risk of heart failure. The two interventions would be performed for acute coronary syndrome and shockable rhythms. In addition, the two interventions can directly achieve reperfusion of the coronary artery and control life-threatening arrhythmias, which is beneficial for restricting the infarction area and stabilizing the hemodynamics. However, patients presenting with cardiac arrest might have severe myocardial damage that is not completely reversible, thus reducing the efficacy of emergent PCI and defibrillation. In addition, although some studies have demonstrated that hypothermia might benefit patients by decreasing infarction area resulting from ST-elevation myocardial infarction (STEMI) in the early post-PCI period (i.e., the first 3 days) [31–33], in this study, only ECMO significantly decreased the risk of heart failure. We suspect the major reason was that ECMO would provide higher chance (or more time) to handle cardiac diseases (i.e., ACS or acute myocarditis) [34, 35]. Once the cardiac diseases were well treated, ECMO could be thought to reduce the risk of following cardiac complications (including heart failure) [36]. Further large randomized controlled trials should be conducted to demonstrate this therapeutic effect of decreasing the risk of new-onset heart failure in OHCA patients. Time-related factors were also considered in this study. We found that most heart failure events occurred within 60 days after OHCA, especially in patients less than 60 years of age. Therefore, early heart function evaluation and treatment should be considered routine for OHCA patients. Finally, prehospital information did not include in this database. Although the associations between prehospital interventions and postresuscitation heart functions had not been clearly addressed, some previous studies pointed out that bystander CPR, public defibrillation were strong predictors of survival (even good neurologic outcome) [37, 38]. Therefore, we reasonably suspect those prehospital interventions would more likely associate with preserved heart functions.

In conclusion, an age from 61–75 years, a history of myocardial infarction or cardiomyopathy, and ischemic heart disease or infection occurring as comorbidities during hospitalization were strong risk factors for new-onset heart failure in OHCA patients. However, ECMO significantly decreased this risk. More importantly, most heart failure events occurred within 60 days after OHCA.

### 4.1. Limitations

We meticulously selected our target patients using specific ICD-9 codes. However, relevant information mentioned in previous studies regarding causes of myocardial dysfunction could not be accessed because this study was based on data from the LHID. Moreover, based on government regulations, the data of patients in small groups based on certain criteria were hidden and could not be analyzed. These are inherent limitations to using the LHID database. However, this is the first study to reveal different degrees of risk for post-OHCA heart failure in different patient age groups. These findings indicate a new direction for studying heart failure after cardiac arrest and possible root causes that need to be clarified. In fact, this database was designed for making national health policy. Therefore, it only included demographics, diagnosis, costs, treatments, and final outcomes. Some detailed medical information (i.e., prehospital factors, findings of echocardiography, and data of recovery) were not available in this database. We hope this article could initially induce the interest of readers in this new topic. Some nature limitations of this study could be handled in our next research project (multicenter studies).

## Figures and Tables

**Table 1:** Characteristics of OHCA patients and comparison patients.

Variables	OHCA patients (*n* = 7,321)		Comparison patients (*n* = 21,963)		*p* value
	No.	%	No.	%	
Sex					
Male	4,528	61.8	13,584	61.8	1.000
Female	2,793	38.2	8,379	38.2	

Age group (y/o)					
<60	2,619	35.8	7,857	35.8	1.000
60–75	1,942	26.5	5,826	26.5	
>75	2,760	37.7	8,280	37.7	

Economic level (monthly income in USD$)^*∗*^					
<600	2,852	39.0	7,281	33.2	<0.001
601–1,000	3,326	45.4	10,330	47.0	
>1,000	1,143	15.6	4,352	19.8	

Geographic region of Taiwan^*∗*^					
Northern	3,480	47.5	10,379	47.3	<0.001
Central	1,812	24.8	5,045	23.0	
Southern	1,747	23.9	5,808	26.4	
Eastern	282	3.9	731	3.3	

Urbanization^*∗*^					
1 (most)	1,722	23.5	5,471	24.9	0.052
2	707	9.7	2,123	9.7	
3	1,812	24.8	5,475	24.9	
4	3,080	42.1	8,894	40.5	

Medical history					
Myocardial infarction^*∗*^	2,251	30.7	2,251	10.2	<0.001
Hypertension^*∗*^	3,960	54.1	5,901	26.9	<0.001
Diabetes mellitus^*∗*^	2,726	37.2	2,498	11.4	<0.001
Cardiomyopathy^*∗*^	44	0.6	12	0.1	<0.001
COPD^a^ and asthma^*∗*^	2,677	36.6	3,122	14.2	<0.001
CKD^b^*∗*^^	1,086	14.8	452	2.1	<0.001
SLE^c^*∗*^^	22	0.3	19	0.1	<0.001
Liver cirrhosis^*∗*^	442	6.0	243	1.1	<0.001
Sicca syndrome^*∗*^	111	1.5	178	0.8	<0.001
Rheumatic arthritis^*∗*^	119	1.6	166	0.8	<0.001

Emergency intervention for OHCA					
Defibrillation^*∗*^	1,255	17.1	4	0.02	<0.001
PCI^d^*∗*^^	532	7.3	29	0.1	<0.001
ECMO^e^*∗*^^	135	1.8	—^f^	—^f^	<0.001
Therapeutic hypothermia^*∗*^	268	3.7	3	0.01	<0.001

New-onset comorbidity during hospitalization					
Infection^*∗*^	1,909	26.1	306	1.4	<0.001
Heart disease					
Ischemic^*∗*^	685	9.4	33	0.2	<0.001
Nonischemic^*∗*^	3,975	54.3	23	0.1	<0.001

^a^COPD: chronic obstructive pulmonary disease; ^b^CKD: chronic kidney disease; ^c^SLE: systemic lupus erythematosus; ^d^PCI: percutaneous coronary intervention; ^e^ECMO: extracorporeal membrane oxygenation. ^*∗*^Statistically significant difference. ^f^Data were not allowed to be displayed if extraction with certain specific parameters resulted in an output of less than 3 patients (identification possible).

**Table 2:** Clinical features of patients who suffered new-onset heart failure.

	Total OHCA patients (*n* = 7,321)		*p* value
	New-onset heart failure		
	Yes (*n* = 363)	No (*n* = 6,958)	
	No. (%)	No. (%)	
Male	210 (57.9)	4,318 (62.1)	0.108
Age group (y/o)^*∗*^			
<60	96 (26.4)	2523 (36.3)	<0.001
61–75	122 (33.6)	1820 (26.2)	
>75	145 (39.9)	2615 (37.6)	
Medical history			
Myocardial infarction^*∗*^	183 (50.4)	2,068 (29.7)	<0.001
Hypertension^*∗*^	230 (63.4)	3,730 (53.6)	<0.001
Diabetes mellitus	149 (41.0)	2,577 (37.0)	0.133
Cardiomyopathy^*∗*^	10 (2.8)	34 (0.5)	<0.001
COPD and asthma	142 (39.1)	2,535 (36.4)	0.314
CKD	64 (17.6)	1,022 (14.7)	0.130
Emergency intervention			
Defibrillation^*∗*^	79 (21.8)	1,176 (16.9)	0.018
PCI^*∗*^	105 (28.9)	427 (6.1)	<0.001
ECMO	5 (1.4)	130 (1.9)	0.687
Therapeutic hypothermia	17 (4.7)	251 (3.6)	0.313
New-onset comorbidity during hospitalization			
Infection^*∗*^	118 (32.5)	1,791 (25.7)	0.006
Heart disease			
Ischemic^*∗*^	92 (25.3)	593 (8.5)	<0.001
Nonischemic^*∗*^	160 (44.1)	3,815 (54.8)	<0.001

^*∗*^Statistically significant difference.

**Table 3:** Covariate-adjusted HRs for new-onset heart failure in different age groups during the 6-month follow-up period.

New-onset heart failure	Total (*n* = 29,284)		OHCA patients (*n* = 7,321)		Comparison patients (*n* = 21,963)	
	No.	%	No.	%	No.	%
All patients	553	1.9	281	3.8	272	1.2
Crude HR^a^ (95% CI^b^)	—		8.8^*∗*^ (7.45–10.5)	1.00		
Age <60 years	—		73	2.8		
Crude HR^a^ (95% CI^b^)	—		5.6^*∗*^ (4.3–7.3)			
Age 60–75 years	—		95	4.9		
Crude HR^a^ (95% CI^b^)	—		11.4^*∗*^ (9.0–14.4)			
Age >75 years	—		113	4.1		
Crude HR^a^ (95% CI^b^)	—		10.7^*∗*^ (8.5–13.4)			

^a^HR: hazard ratio; ^b^CI: confidence interval; ^*∗*^*p* < 0.05.

**Table 4:** Variables influencing the risk of new-onset heart failure according to different age groups.

	Adjusted HRs for new-onset heart failure			
	All patients	<60 years	60–75 years	>75 years
Models and variables	HR (95% CI)	HR (95% CI)	HR (95% CI)	HR (95% CI)
Model 1	8.68 (7.33–10.29)^b^	5.48 (4.22–7.12)^b^	11.20 (8.83–14.20)^b^	10.49 (8.38–13.15)^b^
Comparison patients^a^	1.00	1.00	1.00	1.00

Model 2	5.21 (4.30–6.30)^b^	4.15 (3.16–5.43)^b^	5.36 (4.08–7.04)^b^	4.83 (3.71–6.30)^b^
Comparison patients^a^	1.00	1.00	1.00	1.00
Myocardial infarction	2.47 (2.05–2.98)^b^	2.86 (2.25–3.65)^b^	2.84 (2.24–3.59)^b^	2.38 (1.88–2.99)^b^
Hypertension	1.67 (1.38–2.02)^b^	1.75 (1.38–2.22)^b^	1.61(1.28–2.04)^b^	1.80 (1.43–2.27)^b^
Cardiomyopathy	2.94 (1.45–5.94)^b^	4.62 (1.89–11.29)^b^	1.21 (0.30–4.90)	3.50 (1.11–11.03)^b^
Diabetes mellitus	1.03 (0.84–1.26)	1.02 (0.77–1.35)	1.02 (0.78–1.32)	1.00 (0.78–1.29)
COPD and asthma	1.35 (1.12–1.62)^b^	1.84 (1.46–2.33)^b^	1.68 (1.34–2.11)^b^	1.47 (1.17–1.85)^b^
CKD	1.25 (0.95–1.65)	1.40 (0.91–2.15)	1.24 (0.85–1.82)	1.49 (1.05–2.11)^b^
Liver cirrhosis	0.86 (0.48–1.53)	0.58 (0.24–1.40)	0.74 (0.30–1.78)	1.36 (0.67–2.74)
Atrial flutter/atrial fibrillation	2.02 (1.47–2.78)^b^	3.78 (2.47–5.76)^b^	2.37 (1.56–3.58)^b^	2.40 (1.66–3.47)^b^

Model 3	6.47 (5.34–7.84)^b^	3.13 (2.19–4.74)^b^	7.04 (5.22–9.51)^b^	8.84 (6.91–11.31)^b^
Comparison patients^a^	1.00	1.00	1.00	1.00
Defibrillation	1.36 (1.01–1.83)^b^	1.42 (0.84–2.40)	2.21 (1.41–3.47)^b^	1.03 (0.56–1.87)
PCI	3.95 (3.01–5.18)^b^	5.58 (3.44–9.05)^b^	3.73 (2.41–5.77)^b^	5.78 (3.69–9.03)^b^
ECMO	0.23 (0.07–0.73)^b^	0.40 (0.12–1.29)	—^c^	—^c^
Therapeutic hypothermia	0.87 (0.50–1.53)	1.01 (0.44–2.33)	1.05 (0.46–2.43)	0.56 (0.08–4.03)

Model 4	5.45 (4.27–6.97)^b^	1.57 (0.98–2.50)	4.36 (2.88–6.60)^b^	5.40 (3.74–7.79)^b^
Comparison patients^a^	1.00	1.00	1.00	1.00
Infection	1.53 (1.20–1.94)^b^	3.15 (2.17–4.57)^b^	2.60 (1.80–3.74)^b^	2.34 (1.66–3.31)^b^
Ischemic heart disease	4.50 (3.46–5.86)^b^	6.72 (4.34–10.42)^b^	5.74 (3.80–8.65)^b^	6.39 (4.14–9.85)^b^
Nonischemic heart disease	1.12 (0.88–1.42)	2.16 (1.33–3.51)^b^	1.46 (0.97–2.20)	0.99 (0.68–1.46)
Model 5	3.20 (2.44–4.19)^b^	1.25 (0.77–2.05)	1.92 (1.23–3.00)^b^	2.66 (1.81–3.92)^b^

Comparison patients^a^	1.00	1.00	1.00	1.00

^a^Reference group; ^b^*p* < 0.05; ^c^data were not allowed to be displayed if extraction with certain specific parameters resulted in an output of less than 3 patients (identification possible); Model 1: adjusted by demographics. Model 2: adjusted by medical history. Model 3: adjusted by emergency interventions. Model 4: adjusted by new-onset comorbidities during hospitalization. Model 5: adjusted by all variables (models 1–4).

## Data Availability

This is a national database study. With the government's permission (need proposal), data from the database could be extracted and analyzed for the purpose of scientific research.

## Supplementary Materials

Supplementary Materials Covariate-adjusted HRs for new-onset heart failure in cardiac-caused OHCA patients.
