# Supplementary material for: The Impact of Emergency Interventions and Patient Characteristics on the Risk of Heart Failure in Patients with Nontraumatic OHCA
Source: Emerg Med Int. 2019 Dec 16;2019:6218389. doi: 10.1155/2019/6218389 (PMC6942846; doi:10.1155/2019/6218389)
Supplement: Supplementary Materials — Covariate-adjusted HRs for new-onset heart failure in cardiac-caused OHCA patients. [file 6218389.f1.docx]

**Supplementary Material 1**

Covariate-adjusted HRs for new-onset heart failure in different age groups (for cardiac caused OHCA) during the 6-month follow-up period

| New-onset heart failure | Cardiac caused OHCA patients (n=3,040) | | Comparison patients^a^ (n=9,120) | |
| --- | --- | --- | --- | --- |
|  | No. | % | No. | % |
| **All patients** | 178 | 5.9 | 161 | 1.8 |
| Crude HR ^b^ (95% CI ^c^) | **11.1*(8.9-13.8)** | | 1.00 | |
| **Age <60 years** | 50 | 5.4 |  |  |
| Crude HR ^a^ (95% CI ^b^) | **44.8*(21.2-94.7)** | |  | |
| **Age 60-75 years** | 69 | 7.6 |  |  |
| Crude HR ^a^ (95% CI ^b^) | **15.0*(10.2-22.1)** | |  | |
| **Age >75 years** | 59 | 4.9 |  |  |
| Crude HR ^a^ (95% CI ^b^) | **6.2*(4.5-8.6)** | |  | |

^a^ Matching with acute coronary syndrome, hypertension, atrial flutter/atrial fibrillation and diabetes; ^b^ HR: hazard ratio; ^c^ CI: Confidence interval; * *P*<0.05.
